# A Medical Dictionary for the General Public

**Published:** 1918-08-03

**Authors:** 


### THE INSTITUTIONAL LIBRARY.

### A MEDICAL DICTIONARY FOR THE GENERAL PUBLIC.

This is a medical dictionary* intended for the general public. It is effectively illustrated, and the articles, necessarily brief, are written with much skill. If, as seems to be implied in the Preface, Dr. Drummond has produced the dictionary by the efforts of his own pen, he must be congratulated both on the range of his knowledge and on his capacity to deal with technical subjects in a fashion appropriate to a lay audience. Whether members of the public are really benefited by the reading of medical articles may perhaps be doubted, but there is a natural curiosity in this direction, and out of this demand spring books such as the one now before us. The habit seems to be extending rather than otherwise, and even publications intended for boys and girls produce articles on physiology, with pictures of the bodily organs, and so on; not even the pituitary body escapes popular discussion. If this practice of superficial and arbitrary instruction of the public is to be encouraged, we gladly recognise that Dr. Drummond's book is generally well adapted for the purpose. Here, for example, are provided coloured plates for the diagnostic contrast of the eruptions of scarlet fever and measles; here, too, in black and white, are displayed the intimate windings of the peritoneum; and not less impressive are pictures of the skeleton, the muscles, the nervous system, the heart, the lungs and suchlike mysteries, not to speak of mosquitoes, amœbæ, milking-pails, gas-shades, itch mites, bacteria, starch grains, house-flies, streptococci, rectal syringes, chigoes, breast-exhausters, artificial limbs, thread worms, weighing machines, uric acid, etc., etc. The entertainment, it will be admitted, is a liberal one; of its kind the quality is really excellent; and the price for admission is exceedingly moderate. Dr. Drummond knows quite well that no sensible man will attempt to diagnose or treat disease unless he has had a medical training, and he informs his readers that his book must not lead them to this rash enterprise. Probably his warning will be generally effective, but it is necessary to remember that not all men are sensible, and possibly even not all women. Short of more serious ambitions, the articles are intended "to supplement the doctor's advice" and to enable "his directions to be carried out more faithfully and intelligently." How difficult is the achievement of this aim may be judged from the first item in the dictionary—that is, the paragraph on the "A.B.C. Liniment," where we are told "it must be used with great caution, as it is highly poisonous." Will this information help a mother to carry out the doctor's orders "faithfully and intelligently" when she is advised to apply a liniment of such dramatic possibilities to the sprained ankle of her darling boy? Frankly, we doubt it. The fact is that it is almost impossible to write paragraphs on medical subjects suitable for popular consumption without the risk of misleading the reader, and this danger reaches the highest point when medical treatment is under discussion. Dr. Drummond's volume keeps more clear of this risk than most of his rivals, but neither he nor any other man can avoid it altogether. We repeat our appreciation of the care and ability displayed in his text, and of the effectiveness of his illustrations. Of its kind the book is one of the best we have seen, and this remark applies to its restraint as well as to its positive achievement.

* *A Medical Dictionary.* By W. B. Drummond, M.B., C.M. (London and Toronto: J. M. Dent and Sons, Ltd. 1918. Pp. 625+x. Price 10s. 6d. net.)

### THE AUTHOR'S BOOK HIS EVERLASTING MONUMENT.

"His monument is his work." Men have lavished wealth and labour on great tombs in vain hope to gain everlasting remembrance. The tombs remain or lie in ruins, the inmates are forgotten or remembered only by a few curious. But Colonel William Johnston, C.B., has made for himself a monument that will keep green his memory as long as there is an Army Medical Service. No officer of the Army Medical Service will ever be, but what at some time will at least glance at this great roll and gratefully wonder that any man had the industry and courage to do the work and face the toil. It was courage of a high order that resolved to face the necessary research. It was a marvellous tenacity that held to it till completion. It is regrettable that Colonel Johnston did not live to turn the printed pages of his work and to see that it was good, but the joy is in the chase, not in the end of the run.

The introduction is a concise and interesting history of the Army Medical Service. It tells the story of the great fight for recognition which, won at last, after intense endeavour, with William Johnston in the forefront of the battle, opened the way to the establishment of the Royal Army Medical Corps and to that vast and extraordinary improvement in the *personnel*, in the zeal, and in the scientific work of the Army Medical Service which has resulted in bringing the Service up from a byword for inefficiency to the position of acknowledged excellence it now enjoys. Now it is probably the best State Medical Service in the world, is growing in efficiency, in scientific knowledge, in organising ability, and is the leading authority on tropical diseases and preventative medicine. And William Johnston* lived to see this that he had helped to do, and before he died had the satisfaction of hearing public acclamation of the war work of the Corps he had been so greatly instrumental in bringing to being. His last days must have been filled with satisfaction as he looked on his work brought to fruition, and as he put the final touches to the great roll that was to be posthumously published. The roll has 7,601 names, necessarily the most of them obscure names long forgotten, yet every glance through the pages discovers history and excites fancy. Why is "dismissed" after this name? How was he drowned? "Embarked with a mission to explore the Niger," and so William Cowdery "died at Senegal, December 9, 1815." Where was he when Waterloo was won? Who that has not studied this roll knew that chlorodyne was introduced by an army surgeon; a versatile man, Collis Browne, "invented a scheme for raising sunken vessels, a patent boat propeller, an apparatus for raising and lowering boats, the 'Polyute' shade, and a new theory of wave motion." "Killed by natives of Mozambique." "Killed by Kaffirs." "Killed by an avalanche." Read the record of 4958 James Thomson, and compare it with the record of the German doctors of the years 1914 to 1918. Robert Wilson at Inkerman, Home at Lucknow, Reade at Delhi. Stirring stories briefly told. As a man turns the pages and reads he begins to

* *Roll of Army Medical Services,* 1727-1808. By the late Colonel Wm. Johnston, C.B. Edited by Lieut.-Colonel H. A. L. Howell, R.A.M.C. (Aberdeen: The University Press.)

**The Institutional Library**—*(continued)*.

see what it was that held the man to the work and made it delightful to him. What tales he must have discovered under the dust of musty records! Tales of brave deeds, tales of struggle, tales of foolishness, tales of research, and tales of crime. The man must have had material for a hundred thrilling stories. He could have made a fortune, one would think, had he woven it into romance. But his bent was to the narrow path of research, and his name will live after all living novelists are forgotten.

**The Art of Health.** By James Long. (London: Chapman and Hall, Ltd. 1918. Pp. 192 + ix. Price 5s.)

Mr. Long has the praiseworthy desire to teach his fellows the art of health. His equipment for this task we have to learn from his own pages. There we read that he has been a "life student of the breeding and feeding of domestic animals" and a victim of "poisoned blood . . . colitis, anæmia, and neurasthenia." Under the chastening of these ills he "for four years knew nothing of natural sleep, and none of that peace which passeth all understanding." Fortunately, in the course of his wanderings he met two "happy creatures" who after experiences similar to his own had been restored to their former vigour by forsaking the flesh-pots and holding strictly to a fruit and vegetable diet. Mr. Long followed their example with excellent results, and now desires that all the world should go and do likewise. Hence this book. It belongs to a well-known order. Sometimes the writer is a lady who is specially qualified as the daughter of a former Prime Minister; sometimes it is a gentleman who, having considered the squirrels, finds in these his food exemplar; and sometimes it is the celebrated doctor by whom "cancer . . . is so frequently cured." Mr. Long joins this gallery, but with a somewhat subdued colouring. He is not quite of the stuff of the full-blooded disciple. Thus, on occasions, he admits he has bowed himself in the house of Rimmon, as when, "dining as the guest of the evening at the National Liberal Club," he did not scruple to take "the modest fare . . . including beef," which, it appears, is the note of a great event in that home of political enlightenment but dietetic obscurity. He assures us, however, that this failing was "a mere interlude," and must not be regarded as a serious falling away from the cult of the scraped apple, the grated nut, and the stuffed tomato. Our most deliberate criticism of his book is that it is wearisome, inasmuch as it does little more than repeat what other food faddists have already written. The common programme is strictly followed. Here are the usual pseudo-scientific pretensions, the ordinary charges of medical ignorance, and the complacent personal triumphs of the amateur prescriber, the whole being flavoured with pietistic sentiment and evangelical commonplace. For our own part, and in spite of all this ponderous exhortation and solemn warning, we intend to continue the modest allowance which Lord Rhondda permits, and—we hope for better times!

**The Treatment of Fractures.** By R. Leriche. Edited, with a Preface, by F. F. Burghard, C.B., M.S., F.R.C.S. Vol. I., Fractures involving Joints. Vol. II., Fractures of the Shaft. With illustrations. (University of London Press, Ltd. 1918. 8vo. Pp. xxxiii and 207; pp. xxvi and 305. Price 6s. a vol. net.)

These two volumes are translations of the French edition in the well-known Horizon series. They expound and extend the teaching of the great school of surgery at Lyons where Ollier made his reputation in connection with the diseases and injuries of bone, and Professor Leriche has proved himself a worthy successor. The advice given is clear and practical, for Professor Leriche has himself had such extensive experience in the treatment of war injuries that he has been able to put theory to the test of experiment. He practises conservative surgery, and by conservative surgery he means "the incision of all wounds which experience has shown to be in constant danger of infection: sterilising them by exposure to air, removing as a first step all foreign bodies from a joint and resecting at once any injured joint without waiting for the clinical appearances of articular osteomyelitis." With these principles as a basis he considers in detail the wounds and fractures of the shoulder, elbow, wrist, hip, knee, ankle and tarsus, with a final chapter on multiple wounds of joints. There are numerous illustrations, many of which are radiographs reduced to a diagrammatic form—a useful and somewhat novel idea. The results attained are admirable, but it should be remembered that something is due to a climate, incomparable for the open-air treatment of wounds, in which it has been the good fortune of Professor Leriche to carry out his work. We read with envy of "the truly magical effects of the sun on surface wounds: in a few minutes under one's eyes the bleeding surface is entirely modified. A sitting of a quarter of an hour suffices to get rid of the sloughs, and after three or four sittings suppuration is at an end."

The second volume deals with fractures of the shaft, and Professor Leriche advocates wholeheartedly the removal of every fragment of a shattered bone after careful separation of the periosteum, in opposition to the school of surgery which endeavours to save such fragments in the hope that they will eventually be incorporated by the process of repair. The general principles to be followed in the treatment of fractures is first considered, and then the fractures of individual bones in detail. The two volumes are valuable as an indication of one method of treating fractures, but they do not tell the whole story nor do they indicate the more common methods in use. They are to be looked upon therefore as a valuable but not as a final contribution to one of the most difficult parts of surgery. Mr. Burghard contributes an editorial preface to each volume. The translation is well done, but no index is provided.

**Military Medical Manuals: Malaria in Macedonia.** By P. Armand-Delille, P. Abrami, G. Paisseau, and Henri Lemaire. (University of London Press, Ltd. 6s. net.)

When a disease is much varied in its clinical manifestation and defies treatment it invariably rouses the curiosity of the scientific, stirs many to research, causes controversy and difference of opinion. When in addition the disease is of the most enormous importance, economically, to mankind, and widespread over more than half the world, so much so that perhaps half mankind suffers from it, none can wonder that it has an enormous literature.

Who can read and digest a tenth of the writings on malaria? And here is another and a good book too. Of course many will disagree with much of it. That must be so. The authors have worked laboriously. The fascinating problem of differentiation by clinical observation has had possession of them. Probably that is insoluble, and only the microscope can be relied on for diagnosis; but the problem will continue to attract, and the work done to solve it will continue to add to knowledge. The other malarial problem, What is the best way to give quinine? is also attempted, and solutions are given. May they prove more satisfactory than their predecessors! The book is worth a place in every medical man's library.

